# Training sports dentists: a role in elite sport and society

**DOI:** 10.1038/s41415-025-9098-0

**Published:** 2026-02-27

**Authors:** Peter Fine, Vijay Mathur

**Affiliations:** 41415549749001 Centre for Oral Health and Performance, UCL Eastman Dental Institute, London, UK; 41415549749002 Centre for Dental Education and Research, All India Institute of Medical Sciences, India

## Abstract

Within the specialised world of elite sports, it is important that specifically well-trained ‘sports dentists' are available to support elite athletes. Their role may be very different to that of the general dental practitioner, as the sports dentist needs to have a clear understanding of sporting dental trauma, prevention of tooth tissue loss, screening of athletes during preparation for major sporting events, and be adaptable to the field-of-play role during sporting events. In addition, they need to fulfil the role of a general dental practitioner as well. The training of these individuals is critical to establish a good rapport with the athlete and their supporting medical team. The sports dentist needs to be adaptable as a teacher to other medical specialists, who may not know or have as much experience of what a sports dentist can do. In addition, sports dentists need to understand research protocols to help advance our current knowledge through novel research. A degree-based training programme, particularly the research element, is important for leadership skills and development of sports dentistry.

## Key points

- Training sports dentists can be a complex task depending on the level of education needed. There is a differentiation between those dentists that simply see the occasional athlete during their routine practising lives and those that want to be able to support athletes in their pursuit of sporting excellence.
- Sports dentists working ‘pitch-side' during major sporting events need additional skills, knowledge and confidence.
- Sports dentists can be invaluable to the medical team in several ways: planning manpower for future sporting events, supporting the medical field of play team, and undertaking essential research.
- The importance of modular degree-based training is reflected in the development of leadership skills, knowledge and research for future teaching/learning.

## Introduction

Following the mandatory training of dentists in the United Kingdom (UK) and worldwide, undergraduate dental students must attain a certain standard of care to satisfy the local registration body, for example, in the UK, the General Dental Council. The need for lifelong learning^1^ and the importance of continuing professional development (CPD) in all professions has been well-established.^2^^,^^3^ Many dentists seek further training in aspects of dental treatment that is of particular interest to them. Traditionally, dental specialties such as periodontology, endodontics and prosthodontics have attracted dentists who wish to develop knowledge and skills in these specialties on either a part-time or full-time basis. Added to this are numerous CPD courses that are extremely popular as dentists can choose a topic that they are interested in learning more about without committing to a potentially arduous programme of study.^4^^,^^5^ Traditionally, the training of dentists who care for athletes, whether elite or recreational, involved dealing with dental trauma and how to prevent it.^6^^,^^7^ This role has now evolved into a more holistic approach to caring for athletes. With the acceptance of sports dentistry as an integral part of sports medicine, the role of the sports dentist has expanded to include: screening athletes for oral health issues, being involved in the field of play to support injured athletes, providing comprehensive education to the athletes, and support teams about the impact of poor oral health on athletic performance and the link between oral health and systemic health.^6^^,^^8^ There is also evidence surrounding the impact of fear of re-injury to the facial region on the performance of athletes.^9^ The development of sports dentistry as an integral part of sports medicine has resulted in the need for training of individuals to support athletes in their quest for success.

The role of the sports dentist is ill-defined but can involve the individual in a selection of actions that they may choose to accept or not. Therefore, the training of sports dentists needs to cover a range of options to suit the individual. Gallagher *et al.*, (2021)^6^ reported that the ‘negative impact of poor oral health on athlete-reported wellbeing and performance is clear', emphasising the changing role of the sports dentist including mitigating against the risks to the oral health of elite athletes.

## Manifesto for a training programme

It is clear that the level of training needed to look after recreational athletes on an occasional basis is very different to those skills required to attend to elite athletes with orofacial injuries at major sporting events. It is important to establish what training and the levels of training needed related to the needs of the athletes. A general dental practitioner looking after the weekend warriors from their local soccer team does need to have a working knowledge of dealing with fractured/luxated teeth, which might appear in the practice on Monday morning. This level of knowledge can normally be achieved through CPD courses, perhaps with a hands-on element.

With respect to elite sport, the ‘sports dentist' should have a higher level of knowledge as they will be involved as part of the sports medicine team in providing oral health advice, undertaking dental screening, providing immediate field-of-play care for orofacial trauma, and making decisions about whether an athlete can return to the game after an orofacial trauma. Therefore, training courses need to have certain fundamental elements in place to ensure competence. These include: i) teaching of facial anatomy; ii) an understanding of head injuries; iii) splinting luxated teeth (including avulsions); iv) a keen restorative knowledge; v) awareness of soft tissue injuries and their repair; and vi) preventive methods. Sports dentists can be trained to assess the impact of head injury on the field (similar to a team physio) and can refer for further management to more experienced medical colleagues. There is currently no accreditation system in place to ensure the ongoing standard of care provided by these individuals. However, training courses and programmes are available for interested individuals. For example, in the United States, the annual meeting of the American Sports Dentistry Association is preceded with a day of training; in the UK, the newly formed Sports Dentistry UK Society has a pledge to provide ongoing training for its members and of course formal degree-based learning is now available for interested individuals. There is no mandatory training at the elite sports level at present or any required accreditation to offer dental support to athletes, but it would be desirable to set up some form of annual certification following appropriate training to those individuals caring for elite athletes. Not all dentists will need to be accredited to care for athletes, but professional sport will continue to expect those dentists looking after elite athletes to be skilled, knowledgeable and caring. A pathway of CPD of sports dentists is essential and has been started by Sports Dentistry UK.

## Teaching methods

The teaching of sports dentists necessarily involves practical skills-based teaching sessions, as well as didactic teaching and ideally involves working alongside experienced practitioners that can pass on their knowledge and skills, so called ‘situated learning'.^10^ However, before going out into the sporting arena to work as a sports dentist, individuals need to have a basic knowledge of orofacial trauma, prevention techniques, and screening protocols, including the use of commonly used clinical indices, such as the International Caries Detection and Assessment System,^11^ Basic Erosive Wear Examination,^12^ and Basic Periodontal Examination.^13^ They also need to know how to deal with soft tissue injuries which are reported to be more prevalent than hard tissue injuries,^14^^,^^15^ and be skilled at administering emergency dental treatment in the field of play, so that the injured athlete may be able to return to the match, if appropriate. The literal Latin definition of the word ‘doctor' is ‘teacher' and so sports dentists need to be able to teach, whether that is athletes, coaches, managers, schoolteachers, or medical colleagues. The approach to teaching may vary depending on the cohort being taught and so the sports dentist needs to be familiar with teaching protocols and pedagogy. There can be a possibility of virtual teaching with or without proctored assessment. In addition, discussion forums should be considered after a virtual teaching session.^16^

**Fig. 1:**
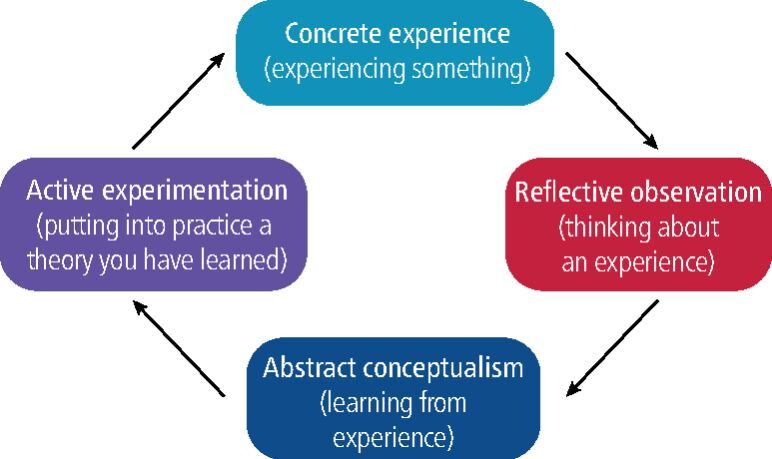
Kolb's reflective/learning cycle

Teaching of skills can take place as CPD courses, as occasional lectures (for example during a conference), as teaching sessions with skilled sports dentists, or by adopting a structured programme leading to a degree-based qualification. The trainee sports dentist needs to be able to reflect on the teaching/learning that they have experienced and by so doing develop their own knowledge and competence through experience.^17^ Kolb developed this model which highlighted the importance of ‘experiential leaning', which has been applied to learning in medicine, dentistry and several other professions (Fig. 1). The pedagogy of teaching sports dentistry skill may involve all four stages, namely didactic learning, learning by observing, learning by assisting and finally, learning by doing.

**Fig. 2:**
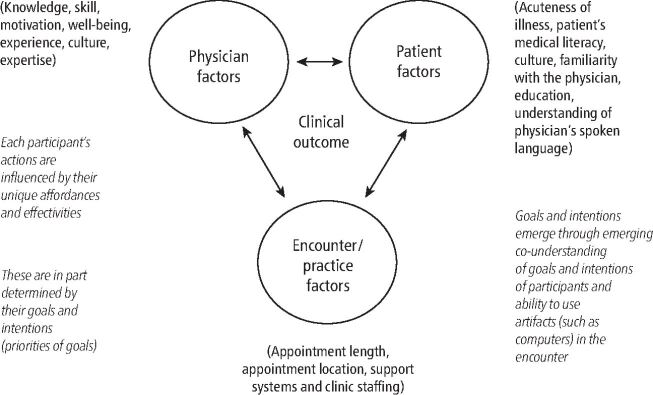
Situated cognition (bold), ecological psychology (italics), distributed cognitions, and the clinical encounter. Reproduced with permission from Durning *et al.*, ‘Situativity theory: a perspective on how participants and the environment can interact: AMEE Guide no. 52', *Medical Teacher*, 2011, reprinted by permission of Taylor & Francis Ltd^23^

It should be remembered that those individuals wishing to learn more about sports dentistry are adults and therefore, their learning pedagogy should reflect this. Learning includes the gaining of three domains: knowledge, skills, and attitudes, so learning should account for each of these domains.^18^ There are broad categories defining adult learning: i) instrumental learning theories, focusing on individual experience, and include the behaviourist and cognitive learning theories; ii) humanistic theories promoting individual development which are more learner-centred to produce individuals who have the potential for self-actualisation, and who are self-directed and self-motivated; iii) transformative learning theory which explores the way in which critical reflection can be used to challenge the learner's beliefs and assumptions;^19^^,^^20^^,^^21^ and iv) social theories of learning, including context and community^22^^,^^23^ (Fig. 2). These concepts have been developed by Etienne Wenger,^10^^,^^24^ who emphasises the importance of ‘communities of practice' in guiding and supporting the learner.

The work by Dunning and Artino (2011) (Fig. 2) illustrates how three factors – physicians (dentists), patients and encounters – influence one's knowledge and therefore the desire to learn. By setting realistic goals as a learner, one can gain knowledge, skill and confidence in clinical procedures.^25^

However, it should be appreciated that adult learning is a different concept to the learning of a school child. Adults who have made a conscious decision to return to postgraduate education have different requirements to a school child (Vygotsky, 1962).^26^ Their decision is based on a realisation that they need more knowledge/skills, they want to learn, and they recognise a desire for specialisation.^27^^,^^28^

As knowledge about sports dentistry and indeed, sports medicine, evolves, it will become even more essential that sports dentists or dentists looking after athletes should be prepared to continually update their knowledge and skills. It is also apparent that the skills needed to be a sports dentist are unlikely to be needed every day in the dental surgery environment. The occasional trauma case will present itself in general dental practice,^29^ so knowledge and skill need to be continuously revised so that when a case does appear, either in the dental setting or in the field of play, the individual is prepared to undertake contemporary treatment that is evidenced-based.^30^

## Degree-based programmes

Currently, there is only one degree-based programme that teaches sports dentists, and this is based at University College London (UCL) Eastman Dental Institute. This three-year part-time programme was developed from a previous CPD course and has enabled teachers and students to explore beyond the basic aspects of dealing with orofacial trauma by including modules on prevention; restorative dentistry; dealing with severe erosive tooth wear cases; oral health and performance; and dealing with facial trauma in the field of play (Table 1). The programme is delivered as a blended learning experience and attracts students globally. All students attend UCL Eastman Dental Institute for one week a year to learn practical skills. The online aspects of the teaching means students can access lectures and seminars when they have time; although, the robust assessment criteria do mean that modules must be completed on time and the module assessment passed before progressing. The final year involves a novel research project that the students undertake having had teaching on setting up a research project, statistical data analysis and dissertation writing. The research module helps clinicians to be better able to assimilate new research and therefore promote an evidence-based approach. Topics covered so far include looking at the position of the mandible while wearing a mouthguard on VO_2_ max (maximal oxygen consumption) levels, the role of the sports dentist within the sports medicine team, the oral health of paralympic athletes and their perceived impact of oral health on athletic performance, whether mouthguards do prevent concussion, and oral health screening of athletes. Publication of these research projects has resulted in advancing the field of sports dentistry. Such training is important for future leadership and development of sports dentistry.

**Table 1:** Sample of modular teaching during the three-year Master's degree programme

**Year**	**Module**
Year 1	Foundations of sports dentistry Orofacial trauma in sport Prevention of sporting dental injuries The wider role of the sports dentist
Year 2	Advanced restorative dentistry Paralympic sports dentistry Dental screening
Year 3	Original research project Statistical data collection and analysis Dissertation writing and viva preparation

Sports dentists wishing to take part in this style of CPD need to be motivated, dedicated and willing to sacrifice time and energy to the programme. Study groups or specialised professional associations may be very helpful in keeping such motivation by organising events and activities.

## Ongoing training/education

Dentistry is evolving rapidly and the need to maintain contemporary knowledge and skills is essential. Therefore, CPD courses are critical to continually update dentists in general, and sports dentists in particular, on new techniques, new evidence and new materials. The use of regular workshops and conferences is also essential as the skills used to support the athletes may not be used every day and so need to be regularly updated and practised, so that the individual does not de-skill. While CPD for sports dentists is not mandatory, it would seem logical that the individuals who are volunteering frequently should keep their skills and knowledge up to date. This is true of those sports dentists who work in their own dental surgery and are happy simply treating local athletes, as well as those individuals who attend sporting events and as members of a sports medicine team who are constantly required to attend matches or at the very least, be available on a Saturday afternoon/evening to receive trauma cases in their practices.

There are also a small band of sports dentists who need to educate the future sports dentists in all aspects of sports dentistry. In addition, these people also need to continually revise their teaching techniques, facilitating teaching/learning for the current sports dentists and investigate new teaching options, for example, the use of artificial intelligence.

The prevention of sporting orofacial injuries is fundamental when training sports dentists. Prevention can be taught in two ways: specific prevention requires interventions by the dentist by providing mouthguards or a protective appliance for the orofacial region, and secondly, sports coaches can teach their athletes specific ways to prevent injuries to vital areas of the face during training or competition. The coaches need to be taught by the sports dentist before they can embark on this approach.

## Elite sport's response to trained sports dentists

It is unrealistic to say that professional sports are only employing qualified sports dentists, but it is equally unrealistic not to say that some very fine dentists are looking after elite athletes on a regular basis and with great skill and experience. As the relationship between various sports and sports dentists develops, the governing bodies of individual sports will look to those individuals who are specially trained to look after their athletes, provide dental support to the medical teams, and educate athletes and medics specifically about the link between oral health and athletic performance. Our roles as dental educators with a keen interest in sports dentistry is to offer our services to sports clubs, sporting bodies and individual athletes, and encourage them to use our knowledge and expertise in supporting athletes so that the negative link between poor oral health and athletic performance may be eliminated.

Despite dental trauma being embedded in the Royal College of Emergency Medicine curriculum and the Intercollegiate Surgical Curriculum Programme (in oral and maxillofacial surgery), those sports dentists who have officiated at major sporting events will testify to the lack of knowledge that our medical counterparts often have when it comes to oral health issues in general.^30^ Bahammam (2018)^31^ reported that ‘the majority of ER (emergency room) physicians lack the knowledge needed to manage avulsions cases'. Further evidence about ‘inadequate knowledge of tooth structure' and therefore the ability to deal with dental trauma was evidenced by Yeng, O'Sullivan and Shulruf (2019)^32^ in their literature review. In the UK, Trivedy *et al.* (2012)^33^ highlighted a lack of confidence among ER physicians in dealing with dental trauma cases. In Europe, the knowledge and skills in dental trauma management among German emergency physicians was found to be ‘generally inadequate' and that ‘targeted training courses are necessary to ensure early and adequate traumatic dental injury treatment to reduce the resulting medical and societal costs as much as possible'.^34^ The need to educate our medical colleagues is essential, even if that education is where to find an appropriate dentist to deal with a potentially life-changing situation, such as an avulsion. There is also a case for educating our dental colleagues in dealing with these relatively uncommon occurrences, which present both on the sports fields and in everyday life. The management of traumatised adult teeth can be a formidable challenge for dental practitioners at any level. Like medical emergencies, initial management during the first few minutes of injury can have a profound influence on prognosis of the tooth/teeth^35^ and the psychological effect on the patient,^36^ including social effectiveness, emotional balance and wellbeing. It is important that practitioners understand the basic principles of managing the acute presentations of dental trauma as outlined by the International Association of Dental Traumatology (IADT) guidelines (2020). This lack of knowledge may be due to a scarcity of cases to practice on rather than a curricular omission. The IADT guidelines help dentists (and medics) to diagnose, treat and support patients following a dental trauma. Ongoing learning in the form of CPD courses needs to highlight these issues for medical and dental practitioners alike. The lack of dental practitioners available to deal with sporting dental emergencies out of normal office hours means that these trauma patients will seek advice and treatment from accident and emergency departments of hospitals, where there is not necessarily a dental specialist on call. While dental trauma is rarely life-threatening, the patient's symptoms and prognosis could be more positive if their trauma is dealt with efficiently by a knowledgeable individual.

## Conclusions

The training of sports dentists should include knowledge about orofacial sports injuries, prevention of these injuries, maintenance of restorative work previously undertaken, specific ‘pitch-side' care during competition, referral of injured athletes for in-hospital specialist treatment, rehabilitation, and recovery without anxiety for re-injury.^9^ Ongoing training of sports dentists should be a continuous process, with some structured programmes, as well as continuing professional education. This specialist knowledge and dedication to supporting athletes is attainable by all dentists. The realisation that motivation, a genuine desire to help athletes, and a clear understanding of the specific issues that particularly elite athletes present with are essential for anyone wishing to pursue this topic and support elite and recreational athletes.
